# Gaps in HCV Knowledge and Risk Behaviors among Young Suburban People Who Inject Drugs

**DOI:** 10.3390/ijerph16111958

**Published:** 2019-06-02

**Authors:** John J. Jost, Barbara Tempalski, Tatiana Vera, Matthew J. Akiyama, Aprille P. Mangalonzo, Alain H. Litwin

**Affiliations:** 1 Montefiore Medical Center, Albert Einstein College of Medicine, 111 E 210th St, Bronx, NY 10467, USA; JJOST@montefiore.org (J.J.J.); tvera@montefiore.org (T.V.); makiyama@montefiore.org (M.J.A.); Aprille.Mangalonzo51@myhunter.cuny.edu (A.P.M.); 2 Institute for Infectious Disease Research, NDRI, Inc. 71 West 23rd Street, 8th Floor, New York, NY 10010, USA; 3 School of Medicine, University of South Carolina, Greenville Health System, 701 Grove Road, Greenville, SC 29605, USA; ALitwin@ghs.org; 4 School of Health Research, Clemson University, 105 Sikes Hall, Box 345124. Clemson, SC 29634, USA

**Keywords:** qualitative, HCV, young suburban PWID, risk behaviors, HCV knowledge

## Abstract

*Background:* Hepatitis C virus (HCV) among young suburban people who inject drugs (PWID) is a growing epidemic in the United States, yet little is known about the factors contributing to increased exposure. The goal of this study was to explore and assess HCV knowledge and attitudes about treatment and identify risk behaviors among a cohort of young suburban PWID. *Methods:* We conducted interviews with New Jersey (NJ) service providers and staff from the state’s five syringe service programs to inform a semistructured survey addressing HCV knowledge, treatment, and risk factors among young suburban PWID. We then used this survey to conduct qualitative interviews with 14 young suburban PWID (median age 26 years) in NJ between April and May 2015. Data were analyzed using a modified grounded theory approach and coded to identify thematic relationships among respondents. *Results:* Most participants had substantial gaps in several aspects of HCV knowledge. These included: HCV transmission, HCV symptoms, and the availability of new direct-acting antiviral therapy. Participants also downplayed the risk of past and current risk behaviors, such as sharing drug paraphernalia and reusing needles, which also reflected incomplete knowledge regarding these practices. *Conclusion:* Young suburban PWID are not receiving or retaining accurate and current HCV information. Innovative outreach and prevention messages specifically tailored to young suburban PWID may help to disseminate HCV prevention and treatment information to this population.

## 1. Introduction

Hepatitis C virus (HCV) among people who inject drugs (PWID) is an ongoing epidemic in the United States (US). The incidence of HCV in the US increased nearly 300% from 2010 to 2015 [1]. PWID comprise greater than 60% of all HCV-infected persons in the US, with the prevalence of HCV ranging from 70–90% [1,2,3].

The HCV epidemic in the US has disproportionately affected young adults (aged <30 years). Although persons born between 1946 and 1964 represent most of the chronic HCV-infected population, from 2010 through 2015, the rate of acute HCV increased the highest among young adults aged 20–29 [1,2,3,4,5]. The rapid increase in the number of reported acute HCV cases among young adults has been linked to a pattern of young opioid users transitioning from prescription opioids to more affordable heroin, and ultimately to heroin injection [6,7,8,9,10,11]. Injection drug use (IDU) was the most common risk behavior (73%) among this cohort [4]. Zibbell et al. (2015), [5] in a study to better understand the increased number of acute HCV cases in central Appalachia, found significant increases (12.6%) in the proportion of persons admitted for drug treatment who noted injection as their primary route of administering drugs. They further reported >100% increase in acute HCV among young adult PWID. Additionally, of the 68% (*n* = 836) of cases that included risk factor data in 2014, 84% (*n* = 702) indicated PWID.

The increased incidence of HCV among young adults has been predominately among non-Hispanic white residents of urban and nonurban areas [4,5,11,12,13,14]. Comparing urban and rural areas, the incidence of acute HCV among rural young adults was twice that of urban young adults [4,5,14,15]. Suryaprasad et al. (2014) [14] reported that among young adult persons with acute HCV, 31% resided in nonurban counties and 67% in urban counties. Incidence of reported acute HCV significantly increased by 13% per year, with an overall 170% increase from 2006 to 2012 in nonurban counties (*p* = 0.003).

While research has emerged regarding factors associated with HCV infection among young adults in urban and rural settings [4,5,14,15,16,17,18,19], little is known about the factors contributing to the high rates of acute HCV among young suburban PWID. Even less is known about how to engage this population in HCV testing and treatment and how to address their questions on a public health scale [4,5,20].

Given the lack of research regarding the perceptions of young suburban PWID toward HCV, it is important to assess this group’s HCV knowledge and how it may impact injection practices, risk behaviors, and the likelihood of seeking HCV treatment if they tested HCV positive. Our study focuses on New Jersey (NJ), where HCV has also been shown to be highly prevalent in young suburban heroin users, including those attending an acute detoxification program. Our study findings suggest that NJ may be experiencing a second wave of HCV infection [21]. The Centers For Disease Control and Prevention (CDC) reports indicate that NJ is included in the eleven states that account for 67.6% of the chronic HCV case reported in the US [1]. As such, the goal of this study is to explore the range of HCV knowledge and reported risk behaviors within a cohort of young suburban PWID in NJ, a previously understudied vulnerable population.

## 2. Methods

We developed an interview guide based on feedback from NJ service providers and the directors of the state’s five syringe service programs (SSPs) regarding young suburban PWID HCV-related issues. These key informants emphasized the lack of HCV knowledge among this young suburban cohort, particularly regarding transmission and treatment. They further noted that these young suburban PWID were not responsive to their offers of HCV testing or HCV treatment referrals. Authors tested the interview guide by conducting preliminary qualitative interviews with 7 young former PWID (age 24–32; women (*n* = 3), men (*n* = 4); all non-Hispanic white) who were currently patients at a residential treatment and recovery program located in Newark, NJ. All had lived in self-identified suburban areas of NJ prior to entering the program. Suburban participtants were identified based on: (1) Residing zip code at time of last recorded risk behavior; and (2) if they lived 10 miles beyond the central city. Each person was asked to discuss their HCV knowledge past drug use and suggested topics to raise with current young PWID. These interviews were not recorded. Detailed notes were taken. All participants for these preliminary interviews agreed to be interviewed after being informed that compensation for their time was not available.

Informed by the preliminary responses and post-interview comments, our interview domains included: Knowledge of HCV transmission, risk behaviors, harm reduction practices, symptoms, long-term effects, and treatment. We also included questions about drug use history, barriers to care, and whether one would seek treatment if tested positive for HCV. We then interviewed a convenience sample of 14 young suburban PWID who were accessing a SSP in Newark, NJ between April and May 2015. In addition to new syringes, the SSP offers HCV, HIV, and STI testing to all individuals accessing the SSP. Inclusion criteria were: (1) Age 18–33; (2) active PWID; and (3) self-identified and currently or recently residing in suburban areas of NJ. The study was described to respondents as a qualitative interview focusing on drug use and HCV knowledge. Interviews were conducted in a private office within the SSP. Participants at SSP were paid $20 for completing the interview. Interviews lasted 40 to 60 minutes. Twelve of the interviews were recorded and professionally transcribed. Extensive notes were taken for the remaining two interviews. At the conclusion of the interview, HCV information was reviewed, including the importance of early detection, the potential absence of clear symptoms, current treatments, and available services.

Transcripts and interview notes were analyzed using a modified grounded theory approach, which calls for simultaneous collection and analysis over the course of data collection [22]. A preliminary list of general, descriptive codes was generated to identify emergent themes, key words, and phrases from early readings of the transcripts. For example, codes related to risk behaviors (e.g., syringe sharing, used syringes) were created, as well as codes related to barriers to HCV treatment (e.g., treatment knowledge, treatment misconceptions). We then independently coded the first four transcripts using Dedoose software and compared coding discrepancies. We developed a codebook that included definitions and examples to ensure that the codes were applied consistently. Codes were added, modified, and eliminated based on feedback from all members of the research team until a consensus was reached [23]. We conducted ongoing comparative analysis to identify emergent themes in regular team meetings. When we reached thematic saturation at 14 interviewers, we ceased interviewing new participants. All names reported in the text are pseudonyms. Prior to the start of each interview, all participants completed a written consent form approved by Albert Einstein College of Medicine’s institutional review board, who approved the study protocol in 2015 (IRB number 2014-3743).

## 3. Results

### 3.1. Participant Characteristics

The majority of participants were male (*n* = 9) and identified as non-Hispanic white (*n* = 11). Their ages ranged from 23 to 33 (median = 26 years). Educational level varied from some high school (*n* = 3) to high school graduation (*n* = 5), GED (*n* = 1), associate degree (*n* = 1), and some college (*n* = 4). None of the participants had completed a bachelor’s degree and two were missing educational information. An equal number were employed (*n* = 6) and unemployed (*n* = 6), with two participants missing information.

All participants reported initiating recreational drug use before the age of 17 (age range 7–16). Half started with alcohol, over a quarter with marijuana, and nearly a quarter a combination of alcohol, marijuana, and/or tobacco. Half moved on to engaging in prescription opioid use during their teenage years (age range 13–18) and half between the ages of 20–28. All had transitioned to heroin, with most sniffing prior to injecting. Thirteen stated that they had acquired a dependence to prescription opioids before moving to heroin, which was cheaper. Age at first injection ranged from 15–28 (median = 23 years). Half reported sharing syringes. All 14 participants reported ever reusing their own syringes. Ten had been tested for HCV, with two testing positive.

### 3.2. Assessing HCV Knowledge and Attitudes, and Identifying Risk Behaviors

The goal of this study was to explore and assess HCV knowledge and attitudes about treatment and identify risk behaviors among a cohort of young suburban PWID. Here we identified two major themes in discussing HCV knowledge among this group: (1) Substantial gaps in knowledge and misinformation regarding HCV, and (2) intent to pursue treatment if tested positive for HCV. Most participants were aware that HCV is a blood-borne virus that affects the liver. However, they had only minimal knowledge regarding transmission, symptoms, and currently available direct-acting antiviral (DAA) medications. Not surprisingly, their responses often included doubts and questions, reflecting their uncertainty. Lastly, we discuss risk behaviors idenified by our survey participants.

### 3.3. Gaps in Knowledge and Misinformation

Transmission: The majority of participants referenced blood and syringe sharing as a means of transmission with a fair amount of certainty. Some additionally noted sex, either directly or by referring to body fluids, semen, and saliva. Patti, 27, who first injected at 18, stated, “You can get it from sharing needles, using it after someone else used it, contact with someone’s blood. I think sexually, as well, but it’s hard to contract that way.”

In some instances, participants professed inaccurate knowledge regarding HCV transmission. Ron, 25, who had been injecting heroin for six months, downplayed blood transmission and emphasized less common ways of contracting HCV. He said:

“Everybody tries to say, ‘Hep C is blood transmitted and that’s the only way you can catch it, [that] you can’t catch it from sex or kissing.’ I have to say that’s a crock of shit. You could have a cold sore in your mouth or a toothache and sore gums. Well, that’s going to lead to bleeding and irritations. With the vagina you could have a yeast infection or any type of irritation that’s gonna cause a flare-up and that’s gonna pass it along…but I believe if you’re someone with Hep C you can possibly pass it just as easily as handing somebody something, you know.”

Some participants believed that it was possible to contact HCV without sharing syringes. Carla, 24, said, “I know that I can get it from myself, if I like have a dirty, like a needle that has blood in it, or microscopic blood that is—”

Jackie, 25, whose first injection was drawn from a shared cooker, believed she contracted HCV via her own “contaminated blood” in a syringe she reused. She said:

“I reused them—that was a mistake that I made. I actually reused them a few times, and then I fucking just found out that I had Hepatitis from using my own needles… I cleaned them out, but I guess from—in the top—the needle, and then there’s a little, where you pull it up, I guess that part had blood in it or something, and I didn’t notice.”

Ivan, 27, took the notion of contracting HCV without sharing a syringe one stop further, stating, “But from what I’ve been told you supposedly can create Hep C on your own”.

Erica, 28, wondered if the length of time of sharing syringes increased her likelihood of contracting HCV. She had been sharing and reusing syringes with her boyfriend for three months. The day before she was interviewed, he revealed that he had been sharing syringes with other people as well and recently tested HCV antibody-positive. She said, “I’ve been with him for five months, and we’ve been sharing needles—well, I’ve been sharing needles with him since February. What’s the chance that it’s—that strong—?”

Symptoms: While the majority of our participants were able to identify blood, mostly via syringe sharing, as the primary means of HCV transmission, they were far less aware of the symptoms and long-terms effects of HCV. Most noted that HCV affects the liver, such as it “kills”, “eats”, or “attacks” the liver. One mentioned liver cancer and cirrhosis.

Few participants were able to point to symptoms, and most of those comments were general and vague, such as “sharp, shooting pain”. Jim, 30, said, “Like it kind of like ruins, hurts on the like the insides and stuff like that, you know?” Only two referred to jaundice, both without specifically naming it. Paul, 30, speculated, “I think one of the symptoms is like your eyes kind of turn yellow.” Similarly, Lynn, 33, stated, “Your skin starts turning yellow and stuff, your teeth.”

DAA knowledge: Nearly half (*n* = 6) of our participants stated that they were unaware of existing DAA HCV treatments and had no comments on the topic when asked about current treatments. Those who were aware that treatment exists where either unable to offer specifics or referred to treatment regimens (Interferon) prior to current DAAs. Doug, 23, said, “I just know it’s medication. I’m going to guess they’re going to treat it like anything else, right? Give you some pills and tell you to be more careful.” Jake, 25, described Interferon-related side effects without specifically mentioning the drug:

“I don’t know the exact name of the medication, but I know that people who have been on the medication, they say it feels like you’re like burning up, or like you have like a fever or something like that. And a lot of them forgo the treatment because of the side effects from the medication.”

Erica spoke of being more hopeful of surviving HCV after conducting an online fact search following her boyfriend’s revelation that he tested HCV antibody-positive but still described incorrect information regarding treatment duration:

“I was like hysterical when he first told me, because I thought it was like a death sentence. I didn’t realize that they have come so far from where they were just ten years ago, you know. So I know now that maybe your body can get rid of it. I think they said like 15% of people will—bodies get rid of it. If not, you just have to be on pills for your whole life, but it’s something you can live with now, possibly.”

Paul was the only participant to refer to newer DAAs, having recently seen a Harvoni commercial: “I know now that they got like some pills you could take and it’s supposed to cure it. At least that’s what they advertise.”

### 3.4. Intent to Pursue HCV Treatment If HCV Positive

Following up on questions regarding HCV treatment, we asked participants if they would seek treatment if they tested HCV positive. Despite having variable knowledge of HCV, all participants stated they would seek treatment if tested positive for HCV. Their responses were short, firm, and succinct. Most emphasized their intentions by saying they would “absolutely”, “definitely” or “for sure” follow up a positive diagnosis by seeking treatment. As Carla, noted, “Hell yes (I would get treatment). I would never kill myself. I’m not suicidal.”

Two participants were less resolute in their intentions to seek HCV treatment, despite their overall intention to do so. Jake, 25, stated, “Depending on what the treatment, how much it costs, but yeah, I would probably try to get treated. It’s the smart thing to do.” Jim, 30, said, “I’d think—yeah. I think so. I wouldn’t want to live with pain and stuff.”

Most participants also stated that the possibility of HCV-related stigma would not influence where they would seek treatment if they tested HCV+. If fact, most said they would prefer undergoing treatment close to where they live. Carla added that she did not have the money to spend on gas to travel to a clinic that was not nearby. Meanwhile, Jake pointed to an additional consideration: “Well, I would probably go to the place that my insurance covers”. And although he said was not worried about stigma related his HCV status, he added, “They’re supposed to keep things confidential and so far, in the past, they’ve been pretty good with that.”

### 3.5. Downplaying Risk Behaviors

The majority of our participants (*n* = 9) reported sharing syringes and/or cookers, cottons, and drug solutions. In reviewing their descriptions of the circumstances surrounding their sharing experiences, we observed a recurring theme of “downplaying” the risks and failing to recognize the possibility of contracting HCV. For example, Ron, who said he only shared syringes with his girlfriend, said, “She got tested last time she was in the mental institution, so that’s why I was like, ‘Okay, I can share with you, because I know we’re both clean.’”

Carla pointed out that she had only shared with a person who claimed to have never shared syringes, noting:

“The only person I shared needles with was this kid that—not that I knew for sure he was clean, but he had just come out of rehab. And no one looks like they have—I don’t know how to explain it. He just was—he never shared needles with anyone before. And because I had just started using, I never had either. So he’s the only person.”

Similarly, Erica felt she was not at risk of contracting HCV because her boyfriend said he only shared with her. Unfortunately, she found this to be untrue. She said:

“He lied to me. He swore, he swore to me on everything that he wasn’t sharing needles. When he called me last night from the detox, he was hysterical crying. And he was like, ‘You need to go to the hospital.’ And I said. ‘what’s wrong, babe?’ And he’s like, ‘You have to get tested.’ And I was like, ‘Tested for what?’ And he said, ‘I just found out I have hepatitis C.’ And I was like, ‘You swore to me you weren’t sharing needles with anybody.’ And he was like, ‘Well, I was sharing needles.’”

In Jackie’s case, she said she has never shared a syringe. However, she did not appear to consider the risk of contracting HCV through sharing a cooker, particularly since she had only done so once. She recalled her first injection:

“By the time I got over there, he had it in the cooker ready, because I thought I wasn’t going [to inject]. And he was like, ‘Well, I got it in the cooker now, so.’ He was like, ‘It’s either you’re going to shoot up, or I can’t give you anything.’ So he had a clean needle, a new needle, so I just tried it out for that one day.”

Miguel, 31, who reuses syringes and cleans them with water, never shared needles. However, he has shared cookers and water and reported that it was happening more frequently: “Now it’s picking up. Like, I will pick up water from the floor if I don’t have it.”

## 4. Discussion

Responses from the participants in this study indicate that this cohort of young suburban PWID in NJ is not receiving accurate information regarding risk of HCV transmission and knowledge of new treatment, such as DAA medications. Most participants minimized reported risk behaviors with respect to HCV, indicating a lack of knowledge regarding HCV transmission. However, despite their gaps in knowledge, all participants stated that they would seek treatment if the tested HCV positive.

Previous research has addressed similar themes identified in this study regarding participants’ lack of HCV basic knowledge, lack of knowledge related to new DAAs, and intent to seek treatment if found to be HCV positive [24]. In addition, active drug use has been noted as a potential barrier to one’s willingness to engage in clinical follow-up after receiving an HCV diagnosis and may further contribute to gaps in HCV information [25,26,27]. In some cases, PWID have reported not receiving clear messages regarding basic HCV knowledge from health providers, such as the meaning of a positive HCV test, the impact of HCV infection, or appropriate next steps.

One of the initial things we observed among our participants was that they did not appear to take advantage of the HCV educational, testing, and medical services offered by the SSP. Most had been tested at some point in the past outside of the SSP, but follow-up testing was not a priority as they continued their injection drug use. This corroborated previous findings in which the effects of testing and counseling were not sustained [18]. However, research has also shown notification of HCV-positive status to be associated with reduced injection drug use [28,29].

Those who rationalized sharing syringes, cookers, cottons, and drug solutions often relied on a significant other or injecting partner to remain uninfected. Such partners were perceived to be at lower risk due to knowledge of this person or the person’s physical appearance [16,19,20]. However, this thought process would only apply to couples who use drugs without a history of injection drug use [30]. In other cases, such rationalizations appear to be cases of misplaced trust. Similarly, respondents rationalized paraphernalia sharing as being a lower risk than sharing syringes, which has been disproved [31].

The report of reusing and not properly cleaning one’s own syringes is also concerning. While not a direct risk for HCV infection, reuse of one’s own syringes can lead to other bacterial infection, such as abscess and endocarditis [31,32,33]. Similarly, improper sterilization is a well-known risk factor for HCV transmission, and PWID should be counseled. PWID should also be made aware that bleach, while more effective, is not 100% effective in preventing transmission but merits consideration for further investigation [34,35].

The stated intent to pursue treatment by all participants if they tested HCV positive was a surprising finding in light of our key informants firmly stating that young suburban PWID are not interested in being tested for HCV or seeking HCV treatment. Key informants had otherwise stated that despite efforts to engage this cohort in testing and other services, young suburban PWID came to SSPs mainly to pick up syringes. Nonetheless, our participants just as firmly stated that they would seek treatment if tested poistive for HCV. However, while these young suburban PWID may intend to seek treatment, intentions do not always lead to actual testing and treatment. PWID have been historically difficult to engage in HCV treatment [28,36,37,38]. In addition, social desirability bias may explain the minimization of risk behaviors and the reported intention to seek treatment if they tested positive for HCV (i.e., the tendency of participants to answer questions in a manner that might be viewed favorably by others) [39]. Previously noted barriers to HCV treatment for illicit drug users should be considered, including competing life priorities due to other comorbidities or to drug use, low perceived vulnerability due to the lack of early symptoms, and fears regarding the side effects of HCV treatment [27,36,37,38]. Stigma had also been seen as a barrier [40] but was not shown by our participants as a barrier for them getting treatment.

This study has several limitations. The participants in this study reflect the current wave of new HCV infections in the United States, which is primarily among young, white, nonurban individuals. However, young PWID of color and sexual minorities were not represented and may evidence different patterns of injection drug use and drug use trajectories. Further, all of our participants owned a car or had available transportation options, which enabled them to access the SSP. Given these limitations, our sample may not have been representative of other young suburban PWID. In addition, the small sample size and absence of follow-up interviews limit the generalizability of our findings. Furthermore, we did not ask our participants how they acquired their HCV information, which would have given us a better idea of how information was received and disseminated. Lastly, despite participants’ high intention to initiate HCV treatment, detailed information on barriers was not elicited and should be further studied.

Despite these limitations, to our knowledge, our study is the first to examine gaps in HCV knowledge and risk behaviors among young adult suburban PWID. This study provides significant insights into how the lack of knowledge and misinformation among this group may contribute to risk behaviors. Future studies should develop and test interventions with larger samples sizes and mixed-methods approaches tailored to this young suburban population, with the aims of providing accurate HCV information which promotes which HCV testing and linkages to HCV care.

Lastly, our findings are similar to a previous study of HCV knowledge among young urban PWID nearly 10 years ago [18]. This suggests that little progress has been made in educating young PWID regarding safe injection practices and HCV treatment, particularly among suburban youth. This is both alarming and surprising, particularly in light of the increasing number of social media campaigns and online health information sites over the past decade [41,42]. This again serves as a call for more effective and innovative efforts to disseminate effective HCV prevention information to this young suburban population.

## 5. Conclusions

The ongoing and increasing rate of HCV infection in suburban areas underscores the need to develop a comprehensive and nuanced understanding of the production of HCV risk and infection among young suburban adults who inject drugs, a population that has been difficult to engage in HCV care. Identifying barriers, facilitators, and service gaps to accessing HCV care and treatment has the potential to address this important public health issue within this understudied suburban cohort. Additional research is needed to understand HCV behavioral risk factors and how to engage and link young suburban PWID to HCV care and harm reduction services in targeted suburban communities in New Jersey.

## References

[1] Centers for Disease Control and Prevention (CDC) (27 June 2018). Commentary|U.S. 2016 Surveillance Data for Viral Hepatitis|Statistics & Surveillance|Division of Viral Hepatitis|CDC. https://www.cdc.gov/hepatitis/statistics/2016surveillance/commentary.htm.

[2] Ly K.N., Xing J., Klevens R.M., Jiles R.B., Ward J.W. (2012). The increasing burden of mortality from viral hepatitis in the United States. Ann. Intern. Med..

[3] Klevens R.M., Liu S., Roberts H., Jiles R.B., Holmberg S.D. (2014). Estimating acute viral hepatitis infections from nationally reported cases. Am. J. Public Health.

[4] Zibbell J.E., Asher A.K., Patel R.C., Kupronis B., Iqbal K., Ward J.W., Holtzman D. (2018). Increases in Acute Hepatitis C Virus Infection Related to a Growing Opioid Epidemic and Associated Injection Drug Use, United States, 2004 to 2014. Am. J. Public Health.

[5] Zibbell J.E., Iqbal K., Patel R.C., Suryaprasad A., Sanders K.J., Moore-Moravian L., Serrecchia J., Blankenship S., Ward J.W., Holtzman D. (2015). Increases in hepatitis C virus infection related to injection drug use among persons aged ≤30 years—Kentucky, Tennessee, Virginia, and West Virginia, 2006–2012. Increases in Hepatitis C Virus Infection Related to Injection Drug Use Among Persons Aged ≤30 Years—Kentucky, Tennessee, Virginia, and West Virginia, 2006–2012. MMWR.

[6] Carlson R.G., Nahhas R.W., Martins S.S., Daniulaityte R. (2016). Predictors of transition to heroin use among initially non-opioid dependent illicit pharmaceutical opioid users: A natural history study. Drug. Alcohol. Depend..

[7] Guarino H., Mateu-Gelabert P., Teubl J., Goodbody E. (2018). Young adults’ opioid use trajectories: From nonmedical prescription opioid use to heroin, drug injection, drug treatment and overdose. Addict. Behav..

[8] Young A.M., Havens J.R. (2012). Transition from first illicit drug use to first injection drug use among rural Appalachian drug users: A cross-sectional comparison and retrospective survival analysis. Addiction.

[9] Lankenau S.E., Teti M., Silva K., Jackson Bloom J., Harocopos A., Treese M. (2012). Initiation into prescription opioid misuse amongst young injection drug users. Int. J. Drug Policy.

[10] Lankenau S.E., Teti M., Silva K., Jackson Bloom J., Harocopos A., Treese M. (2012). Patterns of Prescription Drug Misuse among Young Injection Drug Users. J. Urban Health.

[11] Boodram B., Hotton A.L., Shekhtman L., Gutfraind A., Dahari H. (2018). High-Risk Geographic Mobility Patterns among Young Urban and Suburban Persons who Inject Drugs and their Injection Network Members. J. Urban Health.

[12] Centers for Disease Control and Prevention (CDC) (2011). Hepatitis C virus infection among adolescents and young adults: Massachusetts, 2002–2009. MMWR. Morb. Mortal. Wkly. Rep..

[13] Centers for Disease Control and Prevention (CDC) (2012). Notes from the field: Hepatitis C virus infections among young adults—Rural Wisconsin, 2010. MMWR. Morb. Mortal. Wkly. Rep..

[14] Suryaprasad A.G., White J.Z., Xu F., Eichler B.-A., Hamilton J., Patel A., Hamdounia S.B., Church D.R., Barton K., Fisher C. (2014). Emerging epidemic of hepatitis C virus infections among young nonurban persons who inject drugs in the United States, 2006–2012. Clin. Infect. Dis..

[15] Keyes K.M., Cerda M., Brady J., Havens J.R., Galea S. (2014). Understanding the rural-urban differences in nonmedical prescription opioid use and abuse in the United States. Am. J. Public Health..

[16] Hagan H., Pouget E.R., Williams I.T., Garfein R.L., Strathdee S.A., Hudson S.M., Latka M.H., Ouellet L.J. (2010). Attribution of Hepatitis C Virus Seroconversion Risk in Young Injection Drug Users in 5 US Cities. J. Infect. Dis..

[17] Havens J.R., Lofwall M.R., Frost S.D.W., Oser C.B., Leukefeld C.G., Crosby R.A. (2013). Individual and network factors associated with prevalent hepatitis C infection among rural appalachian injection drug users. Am. J. Public Health.

[18] Jost J.J., Goldsamt L.A., Harocopos A., Kobrak P., Clatts M.C. (2010). Hepatitis C knowledge among new injection drug users. Drugs Educ. Prev. Policy.

[19] Page K., Morris M.D., Hahn J.A., Maher L., Prins M. (2013). Injection drug use and hepatitis C virus infection in young adult injectors: Using evidence to inform comprehensive prevention. Clin. Infect. Dis..

[20] Knight L.L., Wagner K., Leyva Y., Bruce V.R., White K.A.M., Talamantes Y.S., Price B., Page K., Carvour M.L. (2018). Talking about Hepatitis C: FAQs From Young Adults Who Inject Drugs. Health Promot. Pract..

[21] Akyar E., Seneca K.H., Akyar S., Schofield N., Schwartz M.P., Nahass R.G. (2016). Linkage to Care for Suburban Heroin Users with Hepatitis C Virus Infection, New Jersey, USA. Emerg. Infect. Dis..

[22] Corbin J., Strauss A. (2008). Basics of Qualitative Research 3e: Techniques and Procedures for Developing Grounded Theory.

[23] Miles M., Huberman A.M., Saldana J. (2013). Qualitative Data Analysis: A Methods Sourcebook.

[24] Norton B.L., Voils C.I., Timberlake S.H., Hecker E.J., Goswami N.D., Huffman K.M., Landgraf A., Naggie S., Stout J.E. (2014). Community-based HCV screening: Knowledge and attitudes in a high risk urban population. BMC Infect. Dis..

[25] Jones L., Atkinson A., Bates G., McCoy E., Porcellato L., Beynon C., McVeigha J., Bellis M.A. (2014). Views and experiences of hepatitis C testing and diagnosis among people who inject drugs: Systematic review of qualitative research. Int. J. Drug Policy.

[26] Jordan A.E., Masson C.L., Mateu-Gelabert P., McKnight C., Pepper N., Bouche K., Guzman L., Kletter E., Seewald R.M., Des-Jarlais D.C. (2013). Perceptions of drug users regarding Hepatitis C screening and care: A qualitative study. Harm Reduct. J..

[27] Grebely J., Genoway K.A., Raffa J.D., Dhadwal G., Rajan T., Showler G., Kalousek K., Duncan F., Tyndall M.W., Fraser C. (2008). Barriers associated with the treatment of hepatitis C virus infection among illicit drug users. Drug Alcohol Depend..

[28] Mehta S.H., Genberg B.L., Astemborski J., Kavasery R., Kirk G.D., Vlahov D., Strathdee S.A., Thomas D.L. (2008). Limited Uptake of Hepatitis C Treatment among Injection Drug Users. J. Community Health.

[29] Bruneau J., Zang G., Abrahamowicz M., Jutras-Aswad D., Daniel M., Roy E. (2014). Sustained Drug Use Changes After Hepatitis C Screening and Counseling Among Recently Infected Persons Who Inject Drugs: A Longitudinal Study. Clin. Infect. Dis..

[30] Terrault N.A., Dodge J.L., Murphy E.L., Tavis J.E., Kiss A., Levin T.R., Gish R.G., Busch M.P., Reingold A.L., Alter M.J. (2013). Sexual transmission of hepatitis C virus among monogamous heterosexual couples: The HCV partners study. Hepatology.

[31] Pouget E.R., Hagan H., Des Jarlais D.C. (2012). Meta-analysis of hepatitis C seroconversion in relation to shared syringes and drug preparation equipment. Addiction.

[32] Cooper H., Brady J.E., Ciccarone D., Tempalski B., Gostnell K., Friedman S.R. (2007). Nationwide increase in the number of hospitalizations for illicit injection drug use-related infective endocarditis. Clin. Infect. Dis..

[33] Wright N.M.J., Tompkins C.N.E., Jones L. (2005). Exploring risk perception and behaviour of homeless injecting drug users diagnosed with hepatitis C. Health Soc. Care Community.

[34] Kapadia F., Vlahov D., Des Jarlais D.C., Strathdee S.A., Ouellet L., Kerndt P. (2002). Group for the CIDUS. (CIDUS-I). Does Bleach Disinfection of Syringes Protect Against Hepatitis C Infection Among Young Adult Injection Drug Users?. Epidemiology.

[35] Thorpe L.E., Ouellet L.J., Hershow R., Bailey S.L., Williams I.T., Williamson J., Monterroso E.R., Garfein R.S. (2002). Risk of Hepatitis C Virus Infection among Young Adult Injection Drug Users Who Share Injection Equipment. Am. J. Epidemiol..

[36] Treloar C., Newland J., Rance J., Hopwood M. (2010). Uptake and delivery of hepatitis C treatment in opiate substitution treatment: Perceptions of clients and health professionals. J. Viral Hepat..

[37] Litwin A.H., Soloway I., Gourevitch M.N. (2005). Integrating Services for Injection Drug Users Infected with Hepatitis C Virus with Methadone Maintenance Treatment: Challenges and Opportunities. Clin. Infect. Dis..

[38] Stein M.R., Soloway I.J., Jefferson K.S., Roose R.J., Arnsten J.H., Litwin A.H. (2012). Concurrent group treatment for hepatitis C: Implementation and outcomes in a methadone maintenance treatment program. J. Subst. Abuse Treat..

[39] Fisher R.J. (1993). Social Desirability Bias and the Validity of Indirect Questioning. J. Consum. Res..

[40] Treloar C., Rance J., Backmund M. (2013). Understanding barriers to hepatitis C virus care and stigmatization from a social perspective. Clin. Infect. Dis..

[41] Hep C Prevention in Youth. (10 September 2017). https://hepfree.nyc/hep-c-prevention-youth/.

[42] Hepatitis C|Social Media|CDC. (2 April 2013). https://www.cdc.gov/socialmedia/tools/buttons/diseaseandconditions/hepatitis/index.html.

